# Modification of the Susceptibility of Gram-Negative Rods Producing ESβLS to β-Lactams by the Efflux Phenomenon

**DOI:** 10.1371/journal.pone.0119997

**Published:** 2015-03-20

**Authors:** Agnieszka E. Laudy, Paula Osińska, Alicja Namysłowska, Olga Zając, Stefan Tyski

**Affiliations:** 1 Department of Pharmaceutical Microbiology, Medical University of Warsaw, Warsaw, Poland; 2 Department of Antibiotics and Microbiology, National Medicines Institute, Warsaw, Poland; Arizona State University, UNITED STATES

## Abstract

The production of β-lactamases is the most important mechanism of Gram-negative rod resistance to β-lactams. Resistance to ceftazidime and cefepime in clinical isolates of *Enterobacteriaceae* (especially ESβL-positive *E*. *coli* and *K*. *pneumoniae*) and *P*. *aeruginosa* is life-threatening. However, all strains of the above mentioned species possess chromosomally encoded RND efflux pump systems in addition to β-lactamase production. The main goal of this study was to assess the role of efflux pump systems in cefepime and/or ceftazidime resistant phenotypes of ESβL-positive clinical strains of *Enterobacteriaceae* and *P*. *aeruginosa*. The influence of the efflux pump inhibitor PAβN on the minimum inhibitory concentration (MIC) values of tested cephalosporins was species-dependent. Generally, a significant reduction (at least four-fold) of β-lactam MICs was observed in the presence of PAβN only in the case of *P*. *aeruginosa* clinical isolates as well as the ESβL-producing transformant PAO1161 Δ*ampC*. The usage of this agent resulted in the restoration of susceptibility to cefepime and/or ceftazidime in the majority of the *P*. *aeruginosa* ESβL-positive strains with low and moderate resistance to the above cephalosporins. Moreover, an outer membrane permeabilizing effect in the presence of PAβN was identified. Strain-dependent β-lactamase leakage upon PAβN or β-lactam treatment was demonstrated. The most important observation was the restoration of susceptibility of *P*. *aeruginosa* WUM226 to cefepime (MIC decrease from 32 to 4 mg/L) and ceftazidime (MIC decrease from 128 to 4 mg/L) in the presence of PAβN, which occurred despite an almost complete lack of β-lactamase leakage from bacterial cells. In conclusion, these data indicate that RND efflux pumps can modify the susceptibility to β-lactams in Gram-negative rods producing ESβLs. However, this phenomenon occurs only in *P*. *aeruginosa* strains and was not observed among *E*. *coli* and *K*. *pneumoniae* strains, representing the *Enterobacteriaceae* family.

## Introduction

The β-lactams are one of the major classes of antibiotics commonly used in the treatment of infections caused by Gram-negative rods of *Enterobacteriaceae* and non-fermenters. However, the increasing resistance to the third and fourth generations of cephalosporins and carbapenems has become a serious clinical problem worldwide. The primary cause of resistance to these cephalosporins in *Enterobacteriaceae* strains is the production of extended spectrum β-lactamase (ESβL) enzymes [1]. At first, the occurrence of ESβL-type enzymes were most often associated with *Klebsiella pneumoniae* strains, which cause nosocomial outbreaks in intensive care units [2]. At present, various groups of ESβL enzymes are widespread among nearly every pathogenic Gram-negative rod species. However, *K*. *pneumoniae* and *Escherichia coli* are still the most frequently isolated ESβL-producing strains worldwide [1, 2]. Among the Gram-negative non-fermentative rods, *Pseudomonas aeruginosa* is one of the most frequent causes of severe acute nosocomial infections with a 50% mortality rate in chronically ill patients [3]. The resistance of clinical strains of *P*. *aeruginosa* to cephalosporins is most often mediated by the overexpression of the chromosomal AmpC enzyme or by metallo-β-lactamase production [4–6]. However, ESβL-producing strains of *P*. *aeruginosa* are frequently isolated [5, 7–9]. It is important that the presence of different families of ESβL enzymes is described among *Enterobacteriaceae* and *P*. *aeruginosa* strains. These enzymes affect susceptibility to the activity of β-lactamase inhibitors and possibly to the restoration of susceptibility to cephalosporins of isolates belonging to these two groups. Generally, among *Enterobacteriaceae* (e.g., *E*. *coli* and *K*. *pneumoniae*) the most commonly occurring ESβL enzymes are from the TEM, SHV and CTX-M family [1, 2, 10, 11]. However in *P*. *aeruginosa* strains, these types of enzymes are rarely detected [5, 12]. In *P*. *aeruginosa* strains, primarily the ESβL enzymes OXA, PER, GES and VEB-type are detected [5, 7–9].

In the case of *P*. *aeruginosa*, it must be noted that resistance to β-lactams is usually mediated by various mechanisms. Besides the production of inductively expressed natural AmpC β-lactamase, all strains possess chromosomally encoded resistance-nodulation-division (RND) efflux pump systems [4, 5, 13]. Based on the published sequence of the genome of *P*. *aeruginosa* PAO1, it is known that *P*. *aeruginosa* strains may produce 12 different Mex efflux systems from the RND family [13, 14]. As reported, efflux systems like MexAB-OprM, MexXY-OprM and MexCD-OprJ are clinically relevant factors, responsible for decreased susceptibility to β-lactams [13–15]. Moreover, the MexAB-OprM efflux system contributes to both intrinsic and acquired resistance of *P*. *aeruginosa*, while the other two efflux systems participate only in acquired resistance to β-lactams in clinical isolates [15]. On the other hand, cefepime and ceftazidime appear to be relatively poor substrates for the MexAB-OprM system, as in the case of a deletion strain in which 8-fold and 32-fold reductions in the minimum inhibitory concentration (MIC) were observed, respectively [15]. Cefepime, but not ceftazidime, is also a substrate for the MexXY-OprM and MexCD-OprJ efflux systems [15, 16].

It is worth emphasizing that cephalosporins of the third generation (ceftazidime and cefotaxime) are substrates of the AcrAB efflux pump in *K*. *pneumoniae*. However, *in vitro-*selected mutants overexpressing this efflux system are still sensitive to ceftazidime and cefotaxime [17]. AcrAB-TolC is present in various species belonging to *Enterobacteriaceae*, including *E*. *coli* strains [18]. Recently, a significant relationship between overexpression of the *marA* gene and a higher MIC of cefepime for clinical strains of *E*. *coli* was demonstrated [19]. The transcriptional activator MarA has previously been shown to upregulate the production of the AcrAB efflux system in *E*. *coli* [20]. The presence of a new efflux pump has recently been described: KpnEF, from the small multidrug resistance (SMR) family, which also has the ability to remove ceftazidime, ceftriaxone and cefepime in *K*. *pneumoniae* strains [21].

Therefore, the main purpose of this study was to assess the role of efflux pump systems in cefepime- and/or ceftazidime-resistant phenotypes of ESβL-positive clinical strains of *Enterobacteriaceae* represented by *E*. *coli* and *K*. *pneumoniae* as well *as P*. *aeruginosa* strains. Using Phe-Arg-β-naphthylamide (PAβN), a well-known efflux pump inhibitor (EPI), the levels of resistance to the abovementioned cephalosporins in clinical strains and of the ESβL-producing transformants of *P*. *aeruginosa* PAO1161 were investigated [18].

Moreover, the influence of PAβN and β-lactams on the outer membrane permeabilizing phenomenon, leading to the leakage of β-lactamases, was studied by analyzing their ability to hydrolyze nitrocefin in culture supernatants.

## Materials and Methods

### Bacterial strains and growth conditions

The following standard strains were used in the study: *P. aeruginosa* ATCC 27853, *E. coli* ATCC 25922 and *K. pneumoniae* ATCC 700603. The studies were also carried out on 187 clinical strains of ESβL-positive Gram-negative rods, including isolates of *P. aeruginosa* (n = 73), *E. coli* (n = 59) and *K. pneumoniae* (n = 56). Clinical strains were isolated from different materials from patients hospitalized in Warsaw from 2004 to 2011 (in the case of *P. aeruginosa* strains) and from 2010 to 2013 (in the case of *E. coli* and *K. pneumoniae* isolates) and were identified by routine methods using API tests (bioMérieux). Strains were stored at -80ºC until analysis. Prior to testing, each strain was sub-cultured twice on TSA (bioMérieux) medium for 24 to 48 h at 30ºC to ensure viability.

For construction of the *P. aeruginosa* PAO1161 Δ*ampC* strain, the following strains were used: *E. coli* DH5α, *E. coli* S17–1 and *P. aeruginosa* PAO1161 Rif^R^, a derivative of PAO1 [22]. These strains were kindly provided by Prof. G. Jagura-Burdzy (Institute of Biochemistry and Biophysics, Polish Academy of Sciences, Poland). Bacteria were grown in Luria-Bertani broth medium (LB; Difco) or Luria-Bertani agar medium (LB agar; Difco) at 37ºC with or without antibiotics, as appropriate: 0.15 mg/L ampicillin (Sigma) for *E. coli*, 0.125 mg/L rifampicin (Sigma) and 0.3 mg/L carbenicillin (Sigma) for *P. aeruginosa*. The LB agar used for blue/white colony screening contained 0.1 mM isopropyl-β-D-thiogalactopyranoside (IPTG; Sigma) and 0.04 mg/L 5-bromo-4-chloro-3-indolyl-β-D-galactopyranoside (X-Gal; Sigma).

### Construction of the *P. aeruginosa* PAO1161 Δ*ampC* strain

To construct a mutant lacking the *ampC* gene, PCR primers for amplification of the *ampC* gene flanking regions were synthesized on the basis of the nucleotide sequences of *P. aeruginosa* PAO1 in the *Pseudomonas* genome database (http://www.pseudomonas.com/). The following pairs of primers were used in the study for cloning: ampC1F (5^′^-GGGAATTCATGCGGTCGAAGGAGTCACACAGC-3^′^), ampC1R (5^′^-GGGCTGCAGCAGGCAGGGGAATCTGGTAT-3^′^) and ampC2F (5^′^-GGGCTGCAGAACTATCCCAATGCCGAGCG-3^′^), ampC2R (5^′^-GGGGTCGACGATGCCCAGTTGTTCCTCCA-3^′^). For colony PCR and for sequencing: ampC1sF (5^′^-ATGCGGTCGAAGGAGTCACA-3^′^) and ampC2sR (5^′^-GATGCCCAGTTGTTCCTCCA-3^′^) were used.

The *P. aeruginosa* PAO1161 Δ*ampC* mutant was constructed using the broad-host-rage pAKE600 vector using standard genetic methods such as cloning, transformation and conjugation [22–24]. To confirm the deletion of the *ampC* gene in *P. aeruginosa* PAO1161 Rif^R^ the transconjugant colonies of the Suc^R^ Cb^S^ phenotype were analyzed by colony PCR and by sequencing of the PCR products with the primers ampC2sF and ampC2sR.

### Obtaining ESβL-producing *P*. *aeruginosa* PAO1161 derivatives

Plasmid pWUM623, carrying the gene *bla*_GES-1_ encoding for the ESβL, was isolated from a clinical strain of *P*. *aeruginosa* WUM623 and transformed into competent cells of *P*. *aeruginosa* PAO1161 Rif^R^ and *P*. *aeruginosa* PAO1161 Rif^R^ Δ*ampC* mutant. Selection of transformants was performed on LB agar medium with 16 mg/L ceftazidime. The colonies of putative transformants were analyzed by colony PCR and sequencing with the primers GesF (5′-CGCTTCATTCACGCACTATT-3′) and GesR (5′-CTATTTGTCCGTGCTCAGG-3′), as well as by plasmid DNA isolation.

### Plasmid DNA isolation, enzyme digestion and bacterial transformation

Plasmid DNA was isolated using a commercial Plasmid Mini Kit (A&A Biotechnology). Digestion of plasmid DNA and PCR products with Thermo Scientific FastDigest restriction enzymes was carried out according to the supplier’s guidelines (Thermo Scientific).

Competent cells of *E*. *coli* DH5α and *E*. *coli* S17–1 were prepared by the standard CaCl_2_ method and used for standard cloning procedures [23]. Competent cells of *P*. *aeruginosa* PAO1161 Rif^R^ and *P*. *aeruginosa* PAO1161 Rif^R^ Δ*ampC* were prepared by the MgCl_2_ method and their transformation was acheved using the method of Irani and Rowe [24].

### Sequencing and nucleotide sequence analysis

DNA sequencing was performed at the Laboratory of DNA Sequencing and Oligonucleotides Synthesis, Institute of Biochemistry and Biophysics, Polish Academy of Sciences, Warsaw, Poland. DNA and amino acid sequence analysis was carried out using the Vector NTI Advance^TM^ 11 program (Invitrogen). Sequences were compared to those in GenBank NCBI.

### Antimicrobial agents and efflux pump inhibitors

Four antimicrobial agents: cefepime (Bristol-Myers Squibb), ceftazidime (Polpharma), cefotaxime (Sigma), ceftriaxone (the Institute of Biotechnology and Antibiotics, Warsaw) and ofloxacin (Sigma), as well as EPI Phe-Arg-β-naphthylamide (PAβN; Sigma), were used in the study to determine the MIC values of antimicrobial agents ± EPI. Detection of ESβL production by Gram-negative rods was determined using antibiotic discs from Becton Dickinson. For the selection of transformants and transconjugants during construction of the *P*. *aeruginosa* PAO1161 Δ*ampC* mutant, ampicillin (Sigma), carbenicillin (Sigma), rifampicin (Sigma), isopropyl-β-D-thiogalactopyranoside (Sigma) and 5-bromo-4-chloro-3-indolyl-β-D-galactopyranoside (Sigma) were used.

### Detection of ESβL production

ESβL production was verified on Mueller–Hinton II (MH II) agar plates (Becton Dickinson) using disc diffusion tests. *E*. *coli* and *K*. *pneumoniae* ESβL-positive strains were detected by: (Ia) the modified European Committee on Antimicrobial Susceptibility (EUCAST) double-disc synergy test (DDST) with discs containing cefotaxime, ceftazidime, ceftriaxone, cefepime and amoxicillin with clavulanate [25]; (IIa) the extended Clinical and Laboratory Standard Institute (CLSI) confirmatory disc diffusion test using discs with cefotaxime, ceftazidime, cefepime alone and discs of these cefalosporins with clavulanate [26]. ESβL production by *P*. *aeruginosa* strains was estimated by: (Ib) the extended DDST with discs containing aztreonam, ceftazidime, cefepime and a disc with ESβL inhibitors (amoxicillin with clavulanate or ampicillin with sulbactam or piperacillin with tazobactam) [25]; (IIb) the double-disc synergy test with discs containing ceftazidime, cefepime and a disc with the ESβL inhibitor imipenem [27]; (IIIb) the extended CLSI confirmatory disc diffusion test using discs with cefotaxime, ceftazidime, cefepime alone and discs with these cefalosporins supplemented with clavulanate [26]. In all double-disc synergy tests, clear extension of the edge of the inhibition zone of β-lactams toward the discs with ESβL inhibitors confirmed ESβL-type enzyme production by the studied strains. In the confirmatory disc diffusion test, an increase in the inhibition zone diameter of ≥5 mm (for the discs containing cefotaxime or ceftazidime with clavulanate versus cefalosporins alone) according with the CLSI guidelines or ≥2mm (for a combination disc versus cefepime disc alone) according with BioRad disc certification, was interpreted as a positive result, indicating an ESβL-positive strain [26].

### Determination of the MICs of antimicrobial agents ± the efflux pump inhibitor

The influence of the EPI on the susceptibility of clinical strains to cephalosporins was investigated by determining the MIC values of β-lactams. The MIC values of antimicrobial agents in the presence or absence of EPI were estimated on Mueller-Hinton II (MH II) agar (Becton Dickinson) using double agent dilutions, according to the CLSI guidelines [28]. These MIC determinations were carried out on MH II agar with and without 1 mM MgSO_4_.

According to the CLSI guidelines, the breakpoint indicating susceptibility of *P*. *aeruginosa* clinical strains to cefepime and ceftazidime is ≤8 mg/L; however, for clinical isolates of *E*. *coli* and *K*. *pneumoniae* the breakpoint of cefepime is also ≤8 mg/L, but for ceftazidime it is ≤4 mg/L, and for cefotaxime and ceftriaxone it is ≤1 mg/L [26]. The assay was validated by MIC determination of the selected antimicrobial agents against reference strains (*E*. *coli* ATCC 25922 and *P*. *aeruginosa* ATCC 27853) and compared with the CLSI guidelines [26].

To determine the ability of the strains to remove antimicrobial agents by MDR efflux pumps, the MIC values of antimicrobial agents, with or without the pump inhibitor, PAβN (80 mg/L) were evaluated. At least a 4-fold decrease in the MIC value after the addition of PAβN was considered significant.

The influence of PAβN on the susceptibility to cephalosporins of PAO1161 transformants was performed using Etest strips (bioMérieux) on MH II agar (Becton Dickinson) in the presence or absence of PAβN (80 mg/L), according to the manufacturer’s protocol. The β-lactam MIC determinations were carried out on medium with and without 1 mM MgSO_4_.

### Detection of β-lactamases activity in the culture supernatants

In this study, five *P*. *aeruginosa* strains were used: PAO1161, PAO1161 Δ*ampC*, PAO1161 Δ*ampC* (pWUM623) and clinical isolates of *P*. *aeruginosa* WUM623 and WUM226. These strains were cultivated in MH II broth medium with and without 1 mM MgSO_4_. Furthermore, each strain was cultured in the presence and absence of the PAβN EPI at a final concentration of 80 mg/L. Cultures were incubated for 15 hours with shaking at 35ºC. Then, 0.05 mL of each culture was transferred to 10 mL of the fresh identical medium and incubated further. In addition, 0.05 mL of cultures grown on medium lacking the inhibitor were also transferred to new medium with the inhibitor. After three hours, in the early log phase of cultures, the antibiotics (at a final concentration from 1/4 to 1/8 of the MIC value) were added to induce β-lactamase production. For cultures of *P*. *aeruginosa* PAO1161 and PAO1161 Δ*ampC*, ampicillin was added, but for cultures of clinical isolates of *P. aeruginosa* WUM623 and WUM266 and the transformant PAO1161 Δ*ampC* (pWUM623), ceftazidime was used. After three more hours of incubation, all cultures cultivated with and without antibiotics were harvested. Determination of β-lactamase activity in the supernatants of bacterial cultures was performed using a nitrocefin hydrolysis test [29, 30]. Furthermore, the absorption of supernatants derived from different strains was compared.

## Results

### ESβLs production

The production of ESβL enzymes by all studied clinical strains of *P*. *aeruginosa*, *E*. *coli* and *K*. *pneumoniae* was confirmed by the disc diffusion tests.

### Susceptibility of bacteria to cephalosporins

All studied clinical isolates of *P*. *aeruginosa* were divided into three groups depending on the level of resistance to cefepime. Group A contained 18 strains with cefepime MICs of 16–32 mg/L (low level of resistance), group B contained 13 strains with a cefepime MIC of 64 mg/L (moderate level of resistance) and group C contained 42 strains with cefepime MICs ≥128 mg/L (high level of resistance). These strains were also resistant to ceftazidime with the exception of three strains of group A and one of group C (ceftazidime MIC 8 mg/L). All 73 *P*. *aeruginosa* strains showed the same MIC values for cefepime on MH II with and without 1 mM MgSO_4_. However, in seven of these strains, the MIC values of ceftazidime on MH II were twice as high as on MH II with 1 mM MgSO_4_.

Among the studied ESβL-positive strains from the *Enterobacteriaceae* family, all 56 *K*. *pneumoniae* isolates showed resistance to cefepime, cefotaxime and ceftriaxone; the majority of these strains (50 isolates) were also resistant to ceftazidime. In addition, the majority of the 59 clinical *E*. *coli* isolates were resistant to the abovementioned cephalosporins with the exception of 20 strains, among which 15 isolates were only susceptible to ceftazidime, four were susceptible to ceftazidime as well as to cefepime and one isolate was susceptible to cefepime and cefotaxime. Generally, the majority of the studied *Enterobacteriaceae* strains showed the same susceptibility to cephalosporins on MH II regardless of the presence of 1 mM MgSO_4_. In addition, in five *E*. *coli* and eight *K*. *pneumoniae* strains the MIC values of cefepime on MH II were half as much as those on MH II with 1 mM MgSO_4_.

### The effect of an efflux pump inhibitor on the susceptibility of bacteria to cephalosporins

The susceptibility of ESβL-positive clinical isolates of *P*. *aeruginosa* (73 strains), *E*. *coli* (59 strains) and *K*. *pneumoniae* (56 strains) to cephalosporins in the presence of PAβN, an EPI, was tested. PAβN at the concentration used (80 mg/L) did not inhibit the growth of any of the tested strains on MH II medium with or without 1 mM MgSO_4_. As a control for the presence and activity of efflux pumps in the studied strains, the MIC values of ofloxacin, a substrate of efflux pumps, on medium with and without PAβN were determined.

Generally, but only in the case of *P*. *aeruginosa* isolates, a significant reduction (≥4-fold) in β-lactam MICs in the presence of the EPI was obtained. In all *P*. *aeruginosa* strains of groups A and B, and in 17% of the strains of group C, at least a 4-fold decrease in the MIC values of cefepime and/or ceftazidime was observed (Table 1). Very similar results were obtained by performing tests on MH II medium with 1 mM MgSO_4_. Only single strains of *K*. *pneumoniae* (only in the case of ceftazidime) and of *E*. *coli* (regarding sensitivity to cefepime, ceftazidime and cefotaxime) showed at least 4-fold decreases in the MIC values of these cephalosporins in the presence of an EPI (Table 2). The magnitude of the n-fold reductions in the β-lactam MICs for each group of rods is presented in Table 2. The greatest decreases in resistance to β-lactams were observed for ceftazidime, i.e. a ≥32-fold decrease in the MIC for four *P*. *aeruginosa* strains and a 16-fold decrease for one *E*. *coli* isolate. Furthermore, no significant difference in the level of cephalosporin reduction was obtained on MH II medium with and without 1 mM MgSO_4_.

**Table 1:** The effect of PAβN on the susceptibility to cephalosporins of clinical isolates of ESβL-positive *P. aeruginosa*.

*P*. *aeruginosa* ^a^ (No. of strains)	β-lactams	No. (%) of strains with the decreased antibiotic MICs in the presence of PAβN			
		≥4-fold decrease of antibiotic MICs		restoration of susceptibility to antibiotics^b^	
		MH^c^	MH+Mg^c^	MH	MH+Mg
group A (n = 18)	cefepime or ceftazidime	18 (100)^d^	18 (100)	16 (89)^e^	15 (83)
	cefepime	16 (89)	14 (78)	15 (94)	14 (100)
	ceftazidime	18 (100)	17 (94)	15 (83)	14 (82)
	cefepime and ceftazidime	16 (89)	13 (72)	14 (88)	13 (100)
group B (n = 13)	cefepime or ceftazidime	13 (100)	13 (100)	11 (85)	7 (54)
	cefepime	13 (100)	12 (92)	11 (85)	4 (33)
	ceftazidime	13 (100)	13 (100)	7 (54)	7 (54)
	cefepime and ceftazidime	13 (100)	12 (92)	7 (54)	4 (33)
group C (n = 42)	cefepime or ceftazidime	7 (17)	6 (14)	2 (29)	2 (33)
	cefepime	6 (14)	5 (12)	2 (33)	1 (20)
	ceftazidime	6 (14)	6 (14)	2 (33)	2 (33)
	cefepime and ceftazidime	5 (12)	5 (12)	2 (40)	1 (20)

PAβN-efflux pump inhibitor Phe-Arg-β-naphthylamide at a concentration of 80 mg/L; MH-Mueller-Hinton II agar; MH+Mg-Mueller-Hinton II agar with 1mM MgSO_4_.

^a^ *P*. *aeruginosa* isolates were divided into three groups: group A contained strains with cefepime MICs 16–32 mg/L; group B contained strains with cefepime MIC 64 mg/L; group C contained strains with cefepime MICs ≥128 mg/L.

^b^In the presence of PAβN, the MIC values of cephalosporins were ≤8 mg/L.

^c^Antibiotic susceptibility assays were performed on Mueller-Hinton II agar with and without 1 mM MgSO_4_.

^d^As 100% was the number of strains in each group.

^e^As 100% was the number of strains with an observed ≥4-fold decrease of antibiotic MICs in the presence of PAβN.

**Table 2:** The effect of PAβN on the β-lactam MICs against clinical isolates of Gram-negative ESβL-positive rods.

Bacteria (No. of strains)	β-lactams	No. of isolates with indicated n-fold reduction in β-lactam MICs in the presence of PAβN				
		≥4-fold	≥8-fold	≥16-fold	≥32-fold	≥64-fold
*P*. *aeruginosa* group A (n = 18)^a^	cefepime	16 (14)^b^	5 (4)	1 (1)	0	0
	ceftazidime	18 (17)	11 (10)	5 (4)	1 (1)	0
*P*. *aeruginosa* group B (n = 13)^a^	cefepime	13 (12)	4 (4)	0	0	0
	ceftazidime	13 (13)	11 (11)	6 (6)	2 (2)	0
*P*. *aeruginosa* group C (n = 42)^a^	cefepime	6 (5)	2 (2)	2 (1)	1 (1)	0
	ceftazidime	6 (6)	2 (2)	2 (2)	1 (1)	1 (1)
*E*. *coli* (n = 59)	cefepime	1 (1)	1 (1)	0	0	0
	ceftazidime	1 (1)	1 (1)	1 (1)	0	0
	cefotaxime	1 (1)	0	0	0	0
*K*. *pneumoniae* (n = 56)	ceftazidime	1 (1)	0	0	0	0

PAβN-efflux pump inhibitor Phe-Arg-β-naphthylamide at a concentration of 80 mg/L.

^a^ *P*. *aeruginosa* isolates were divided into three groups: group A contained strains with cefepime MICs 16–32 mg/L; group B contained strains with cefepime MIC 64 mg/L; group C contained strains with cefepime MICs ≥128 mg/L.

^b^In parentheses, the results obtained on Mueller-Hinton II agar with 1 mM MgSO_4_ are presented.

In contrast to the *P*. *aeruginosa* strains, in the case of a significant number of *Enterobacteriaceae* isolates, an increase in resistance to cephalosporins was observed in the presence of PAβN (Table 3). First of all, in a large number of isolates, i.e. for 15 *K*. *pneumoniae* (27%) and 5 *E*. *coli* (9%) strains, at least a 4-fold increase in the MIC value of cefepime was obtained. Moreover, the same strains showed increased resistance to other (one or two) cephalosporins. Antibiotic susceptibility assays were performed on MH II with and without 1 mM MgSO_4_. A difference in susceptibility to cefotaxime in the presence of PAβN was observed in the case of four *K*. *pneumoniae* strains that did not show at least a 4-fold increase in the cefotaxime MIC on medium with 1 mM MgSO_4_ and the EPI.

**Table 3:** The effect of PAβN on the susceptibility to cephalosporins of clinical isolates of ESβL-positive *Enterobacteriaceae*.

Bacteria (No. of strains)	β-lactams	No. (%) of strains with ≥4-fold increase of antibiotic MICs in the presence of PAβN	
		MH	MH+Mg
*E*. *coli* (n = 59)	cefepime	5 (9)	5 (9)
	cefotaxime	2 (3)	2 (3)
	ceftriaxone	3 (5)	3 (5)
	cefepime and ceftriaxone	1 (2)	1 (2)
	cefepime and cefotaxime and ceftriaxone	2 (3)	2 (3)
*K*.*pneumoniae* (n = 56)	cefepime	15 (27)	15 (27)
	cefotaxime	8 (14)	4 (7)
	ceftriaxone	2 (4)	2 (4)
	cefepime and cefotaxime	6 (11)	6 (11)
	cefepime and cefotaxime and ceftriaxone	2 (4)	2 (4)

PAβN-efflux pump inhibitor Phe-Arg-β-naphthylamide at a concentration of 80 mg/L; MH-Mueller-Hinton II agar; MH+Mg-Mueller-Hinton II agar with 1mM MgSO_4_.

### PAβN affects the restoration of β-lactams susceptibility in ESβL-positive rods

Interestingly, as was shown in Table 1, for the majority of *P*. *aeruginosa* ESβL-positive strains of groups A and B, the restoration of susceptibility to cefepime and/or ceftazidime was observed. The MIC values of these cephalosporins decreased in the presence of PAβN and amounted to ≤8 mg/L, which is the breakpoint and indicates susceptibility of *P*. *aeruginosa* strains to cefepime and ceftazidime. The greatest increase in bacterial susceptibility to β-lactams (for an MIC of 1–2 mg/L) was observed in three isolates from group A and in the other three isolates from group B. In the case of four resistant strains, the MICs of ceftazidime were reduced from 16–32 to 1–2 mg/L and the other two strains showed a restoration of susceptibility to cefepime (the MICs changed from 16–32 to 1–2 mg/L). For the strains with moderate or high levels of resistance to cefalosporins (MICs 64–128 mg/L), a decrease in the MICs to values of 4–8 mg/L in the presence of PAβN was observed. It is of little significance that only two isolates among the 42 included in group C showed a restoration of susceptibility to the studied cephalosporins. Moreover, very similar results of susceptibility restoration of *P*. *aeruginosa* strains to cefepime and/or ceftazidime were obtained on MH II medium with 1 mM MgSO_4_, excluding seven strains (54%) from group B. These seven isolates did not show a restoration of susceptibility to cefepime on the medium supplemented with 1 mM MgSO_4_, in comparison to the results on medium without MgSO_4_.

### PAβN reduces the acquired resistance to cephalosporins in ESβL-positive PAO1161 transformants

For the study carried out on laboratory strains of PAO, an ESβL-type enzyme encoded by a plasmid-located gene was selected. Moreover, this ESβL-producing clinical strain of *P*. *aeruginosa* showed increased activity (decreased MIC values of at least 4-fold) against cefepime as well as ceftazidime in the presence of PAβN, compared to its absence, on MH II with or without 1 mM MgSO_4_. The selected *bla*_GES-1_ gene was located on the large plasmid pWUM623 in a *P*. *aeruginosa* WUM623 clinical isolates.

As shown in Table 4, the transfer of plasmid pWUM623 to PAO strains resulted in the transformant’s resistance to both cephalosporins; the MIC values increased from 1.5 to 128 mg/L and above. In addition, in both transformants, i.e. PAO1161 (pWUM623) and PAO1161 Δ*ampC* (pWUM623), ESβL enzyme production was confirmed by double-disc synergy tests. The most important result, for the clinical strains as well as for the studied transformants, was the observed decrease in resistance to β-lactams in the presence of the EPI. In the case of *P*. *aeruginosa* WUM623, the MIC value of cefepime decreased by 11-fold and for ceftazidime decreased by over 11-fold in the medium containing PAβN. However, for both transformants an 8-fold decrease in the cefepime and ceftazidime MIC was obtained. Generally, there were no differences between the results for the PAO1161 and PAO1161 Δ*ampC* strains, regardless of the presence of PAβN or MgSO_4_.

**Table 4:** Influence of PAβN on the increase of susceptibility to cephalosporins in *P. aeruginosa* PAO1161 transformants.

*P*. *aeruginosa* strains	MIC (mg/L)			
	Cefepime		Ceftazidime	
	MH	MH+PAβN	MH	MH+ PAβN
WUM623	128 (128)^a^	12 (12)	>256 (>256)	24 (24)
PAO1161	1.5 (1.5)	0.75 (0.75)	1.5 (1)	0.38 (0.38)
PAO1161 (pWUM623)	128 (128)	16 (16)	>256 (>256)	32 (32)
PAO1161 Δ*ampC*	1.5 (1.5)	0.75 (0.75)	1.5 (1)	0.38 (0.38)
PAO1161 Δ*ampC* (pWUM623)	128 (128)	16 (16)	>256 (>256)	32 (32)

MH-Mueller-Hinton II agar; MH+PAβN-Mueller-Hinton II agar with efflux pump inhibitor Phe-Arg-β-naphthylamide at a concentration of 80 mg/L.

^a^In parentheses, the results obtained on Mueller-Hinton II agar with 1 mM MgSO_4_ are presented.

### Strain-dependence of β-lactamases leakage into the culture supernatant upon PAβN or β-lactam treatment

The effect of the influence of PAβN on outer membrane permeabilization and leakage of β-lactamases into the culture supernatant was determined by the nitrocefin hydrolysis test. For this study, two ESβL-positive *P*. *aeruginosa* clinical isolates (WUM226 and WUM623) were selected. In the presence of the EPI, both strains showed a significant decrease in the MIC value for cefepime (from 32 to 4 mg/L for WUM226 and from 128 to 12 mg/L for WUM623), ceftazidime (from 128 to 4 mg/L for WUM226 and from >256 to 24 mg/L for WUM623) and ofloxacin (from 32 to 0.5 mg/L for WUM226 and from 4 to 0.125 mg/L for WUM623), regardless of the presence of MgSO_4_. However, there was a difference in the permeability of the cell envelope for β-lactamases in these strains (Table 5). In the case of *P*. *aeruginosa* WUM226, cultivation on medium containing the inhibitor for 6 or 21 hours only marginally affected β-lactamase leakage into the supernatant. Moreover, the addition of an antibiotic to the culture did not change the absorbance. In contrast, the cell envelope of *P*. *aeruginosa* WUM623 as well as PAO1161 and PAO1161 Δ*ampC* (pWUM623) exhibited the ability to increase permeability due to the presence of β-lactams or PAβN. Moreover, the level of measured absorbance indicating β-lactamase activity in culture supernatants was higher after 21 hours of cultivation in medium containing the inhibitor than when PAβN was added only during the last 6 hours of incubation. Additionally, the permeability effect of PAβN was partially abolished in the presence of 1 mM MgSO_4_ for PAO1161 and PAO1161 Δ*ampC* (pWUM623) but not for the WUM623 strain.

**Table 5:** The presence of β-lactamases in bacterial culture supernatants as visualized by the nitrocefin hydrolysis test.

Strains	Relative absorbance^a^							
	Without antibiotic				With antibiotic^b^			
	MH	MH+Mg	MH +PAβN	MH+Mg +PAβN	MH	MH+Mg	MH +PAβN	MH+Mg +PAβN
PAO1161	0.008	0.008	0.003^c^ (0.130)^d^	0.002^c^ (0.030)^d^	0.012	0.007	0.029^c^ (0.340)^d^	0.020^c^ (0.176)^d^
PAO1161 Δ*ampC*	0.008	0.006	0.003 (0.007)	0.003 (0.006)	0.009	0.008	0.004 (0.006)	0.005 (0.006)
PAO1161 Δ*ampC* (pWUM623)	0.133	0.117	0.588 (1.450)	0.120 (0.318)	0.253	0.155	0.720 (1.515)	0.240 (0.533)
WUM623	0.101	0.064	0.762 (1.455)	0.637 (1.245)	0.270	0.215	0.755 (1.495)	0.745 (1.430)
WUM226	0.012	0.012	0.028 (0.038)	0.025 (0.036)	0.012	0.012	0.025 (0.050)	0.024 (0.047)

MH-Mueller-Hinton II agar; MH+Mg-Mueller-Hinton II agar with 1mM MgSO_4_; MH+PAβN-Mueller-Hinton II agar with efflux pump inhibitor Phe-Arg-β-naphthylamide at a concentration of 80 mg/L; MH+Mg+PAβN-Mueller-Hinton II agar with 1mM MgSO_4_ and with efflux pump inhibitor Phe-Arg-β-naphthylamide at a concentration of 80 mg/L.

^a^The presence of β-lactamases in bacterial culture supernatants was assessed by measurement of the rates of nitrocefin hydrolysis as relative absorbance at 486 nm.

^b^Antibiotics used for cultivation of *P*. *aeruginosa* strains: PAO1161—ampicillin 50 mg/L in MH/MH+Mg medium and ampicillin 4 mg/L in MH/MH+Mg with PAβN; PAO1161 Δ*ampC*—ampicillin 16 mg/L in MH/MH+Mg medium and ampicillin 3 mg/L in MH/MH+Mg with PAβN; PAO1161 Δ*ampC* (pWUM623) and WUM623—ceftazidime 32 mg/L in MH/MH+Mg medium and ceftazidime 4 mg/L in MH/MH+Mg with PAβN; WUM226—ceftazidime 16 mg/L in MH/MH+Mg medium and ceftazidime 1 mg/L in MH/MH+Mg with PAβN;

^c^The strains were cultivated for 15 hours on a medium without inhibitor and then transferred to new medium containing the inhibitor.

^d^In parentheses, the results obtained for the strains cultivated for 15 hours on medium containing the inhibitor and transferred to the identical medium for further incubation.

## Discussion

It is known that MDR efflux pumps from the RND family play a significant role in the resistance of Gram-negative rods to several antibiotics from different chemical groups, including β-lactams [13, 18]. The main mechanism of resistance to ceftazidime and cefepime of the majority of *Enterobacteriaceae* and *P*. *aeruginosa* clinical isolates is β-lactamase production [1, 2, 5, 13]. ESβL-positive *E*. *coli* and *K*. *pneumoniae* strains remain a global public health problem [1]. Like most *Enterobacteriaceae* rods, *P*. *aeruginosa* strains are also able to produce ESβL-type enzymes [5, 13]. In this study, the role of MDR efflux pumps and ESβL-type enzymes in the resistance to β-lactams in clinical isolates of Gram-negative rods was investigated. Interestingly, only ESβL-positive *P*. *aeruginosa* isolates showed a significant reduction (≥4-fold) in the MIC values for cefepime and/or ceftazidime in the presence of an EPI. Additionally, the influence of PAβN on the reduction in the MIC values of the tested cephalosporins depended of the strain’s resistance level to β-lactams. The most important observation was the restoration of susceptibility to cefepime and/or ceftazidime in the majority of ESβL-positive *P*. *aeruginosa* strains with low and moderate levels of resistance to cefepime. The MIC values of these cephalosporins decreased in the presence of PAβN and amounted to ≤8 mg/L, the breakpoint indicating susceptibility of *P*. *aeruginosa* strains to cefepime and ceftazidime [26]. Our results suggest that MDR efflux pumps are responsible for the low and moderate resistance to cefepime and/or caftazidime of ESβL-positive *P*. *aeruginosa* strains. In contrast, ESβL-type enzyme production was responsible for the high level of resistance to both cephalosporins. Interestingly, this effect was not observed among ESβL-positive *E*. *coli* and *K*. *pneumoniae* strains regardless of the level of resistance to third and/or fourth generation cephalosporins.

In order to explain whether the efflux phenomenon is the main reason for the resistance to β-lactams of *P*. *aeruginosa* strains producing ESβLs, further studies were carried out using *P*. *aeruginosa* PAO1161 transformants. *P*. *aeruginosa* PAO1161 Rif^R^ (leu^-^ r^-^) is a derivative strain of PAO1, for which the complete genome sequence has been published [14]. It is known that *P*. *aeruginosa* strains produce two chromosomally encoded β-lactamases, AmpC and PoxB [13]. The overexpression of naturally occurring AmpC causes a decrease in susceptibility or even resistance to extended-spectrum cephalosporins [5, 13]. In our study, the *P*. *aeruginosa* PAO1161 Δ*ampC* mutant was constructed and used for the transformation of a plasmid carrying the gene coding for ESβL. The plasmid pWUM623 containing the *bla*_GES-1_ gene was selected. In the case of the clinical isolate of *P*. *aeruginosa* WUM623, a decrease in the cephalosporin MIC values was obtained in the presence of the EPI. The transfer of plasmid pWUM623 to *P*. *aeruginosa* PAO strains resulted in a high level of resistance of the PAO1161 (pWUM623) and PAO1161 Δ*ampC* (pWUM623) transformants to both cephalosporins and production of ESβL enzymes.

The most important outcome of this study is that our results confirmed the participation of efflux pumps in the resistance to cefepime and/or ceftazidime of both ESβL-producing transformants. In the presence of PAβN, a decrease in the MIC values for cefepime and ceftazidime was obtained in the case of both transformants. However, some authors have reported that PAβN permeabilizes bacterial membranes in a concentration-dependent manner [31, 32]. Furthermore, PAβN does not affect the proton gradient across the inner membrane [32]. It is known that the RND efflux pumps use the energy of the proton gradient across the inner membrane for drug extrusion. Therefore, the mode of action of PAβN is not based on the deenergization of efflux pumps [32]. So, another important issue to consider for understanding the participation of efflux pumps in the resistance to β-lactams of ESβL-positive strains is to explain the impact of the presence of PAβN on the permeability of the outer membrane of Gram-negative bacteria. This possibility appeared to be feasible on the basis of the structure of PAβN, which is a cationic peptide with two positive charges at physiological pH [31–33].

In a previous report concerning AmpC overexpression by a *P*. *aeruginosa* PAO1 *dacB* mutant strain, leakage of AmpC into the culture supernatant upon PAβN treatment was described [31]. This effect was very weak in the absence of PAβN, increased in the presence of 10 mg/L of the inhibitor for a few hours and was very significant at the 25 mg/L and 50 mg/L concentrations of PAβN. However, AmpC activity was not observed in the culture supernatant of the wild type strain (PAO1), regardless of the PAβN concentrations [31]. Similar results were obtained in this report for PAO1161, but the extension of the cultivation time to 21 hours in the medium with the inhibitor led to a slight increase in AmpC activity in the supernatant. More significantly, β-lactamase leakage into the supernatant in the presence of PAβN is determined by RND efflux pump activity [32]. In the case of the mexAB-overexpressing *P*. *aeruginosa* strain, there was no influence of PAβN at various concentrations (2–128 mg/L) on the passage of AmpC into the culture supernatant. On the other hand, this inhibitor was capable of altering the permeability of the outer membrane of *P*. *aeruginosa* mutants with impaired function of the MexAB-OprM efflux pump [32].

In this report, the outer membrane permeabilizing effect in the presence of PAβN was investigated. For the clinical strain of *P*. *aeruginosa* WUM623 carrying the *bla*_GES-1_ gene and the *P*. *aeruginosa* PAO1161 Δ*ampC* (pWUM623) transformant, which retained the efflux pump activity of the wild-type strain, the presence of β-lactamases in the supernatant was confirmed by the nitrocefin hydrolysis test. GES-1 leakage was observed in the log phase of bacterial growth in the medium without or with an antibiotic; however, the presence of PAβN increased the level of β-lactamase activity in the supernatant.

Previously, it was described that magnesium supplementation, at a concentration of 1 mM MgSO_4_, reduced the loss of intracellular AmpC in the AmpC-overexpressing *P*. *aeruginosa* PAO1 *dacB* mutant and completely protected the outer membrane of the *P*. *aeruginosa* Δ*mexA* mutant against the permeabilizing effect of PAβN [31, 32]. In the current study, it was shown that despite the differences in the influence of 1 mM MgSO_4_ on the protection of *P*. *aeruginosa* WUM623 and *P*. *aeruginosa* PAO1161 Δ*ampC* (pWUM623) cells against β-lactamase leakage, both strains showed a very similar degree of increase in sensitivity to cefepime and ceftazidime in the presence of PAβN. These data indicate that despite the membrane permeabilizing activity of PAβN, the main mechanism of action of this agent involves the inhibition of the MDR efflux pumps in *P*. *aeruginosa* strain. As previously indicated, PAβN inhibits the activity of RND efflux pumps in a competitive manner [18, 31].

Moreover, using the example of two ESβL-producing clinical strains, *P*. *aeruginosa* WUM226 and WUM623, the strain-dependence of β-lactamase leakage into the culture supernatant upon PAβN or β-lactam treatment was shown. It is likely that the resistance of *P*. *aeruginosa* WUM226 to both cephalosporins is the result of overexpression of the mexAB efflux pump. As previously published, the presence of PAβN had no effect on the passage of β-lactamase into the culture supernatant in the mexAB-overexpressing *P*. *aeruginosa* strain [32]. The most important finding is the restoration of susceptibility of *P*. *aeruginosa* WUM226 to cefepime and ceftazidime in the presence of PAβN, which was observed despite the virtual lack of β-lactamases leakage from bacterial cells.

In conclusion, these data indicate that RND efflux pumps can modify susceptibility to β-lactams in Gram-negative rods producing ESβLs, but this phenomenon occurs only in *P*. *aeruginosa* strains and is not observed among *E*. *coli* and *K*. *pneumoniae* strains from the *Enterobacteriaceae* family.

The activity of efflux pumps is responsible for the low and moderate resistance to cefepime and/or caftazidime in ESβL-positive *P*. *aeruginosa* strains. The use of PAβN as an EPI resulted in the restoration of susceptibility to cefepime and/or ceftazidime for the majority of *P*. *aeruginosa* ESβL-positive strains from the abovementioned groups. Considering the results of these studies, the discovery of a compound with EPI activity against RND efflux pump systems will likely be useful as a new therapy for severe infections caused by some ESβL-positive *P*. *aeruginosa* strains.
